# Expandable Drug Delivery Systems Based on Shape Memory Polymers: Impact of Film Coating on Mechanical Properties and Release and Recovery Performance

**DOI:** 10.3390/pharmaceutics14122814

**Published:** 2022-12-15

**Authors:** Marco Uboldi, Chiara Pasini, Stefano Pandini, Francesco Baldi, Francesco Briatico-Vangosa, Nicoletta Inverardi, Alessandra Maroni, Saliha Moutaharrik, Alice Melocchi, Andrea Gazzaniga, Lucia Zema

**Affiliations:** 1 Sezione di Tecnologia e Legislazione Farmaceutiche “Maria Edvige Sangalli”, Dipartimento di Scienze Farmaceutiche, Università degli Studi di Milano, Via G. Colombo 71, 20133 Milano, Italy; 2 Dipartimento di Ingegneria Meccanica e Industriale, Università degli Studi di Brescia, Via Branze 38, 25123 Brescia, Italy; 3 Dipartimento di Chimica, Materiali e Ingegneria Chimica “G. Natta”, Politecnico di Milano, Piazza Leonardo da Vinci 32, 20133 Milano, Italy

**Keywords:** shape memory polymer, poly(vinyl alcohol) (PVA), hot melt extrusion, fused deposition modeling, film-coating, retentive drug delivery system, expandable drug delivery system

## Abstract

Retentive drug delivery systems (DDSs) are intended for prolonged residence and release inside hollow muscular organs, to achieve either local or systemic therapeutic goals. Recently, formulations based on shape memory polymers (SMPs) have gained attention in view of their special ability to recover a shape with greater spatial encumbrance at the target organ (e.g., urinary bladder or stomach), triggered by contact with biological fluids at body temperature. In this work, poly(vinyl alcohol) (PVA), a pharmaceutical-grade SMP previously shown to be an interesting 4D printing candidate, was employed to fabricate expandable organ-retentive prototypes by hot melt extrusion. With the aim of improving the mechanical resistance of the expandable DDS and slowing down relevant drug release, the application of insoluble permeable coatings based on either Eudragit^®^ RS/RL or Eudragit^®^ NE was evaluated using simple I-shaped specimens. The impact of the composition and thickness of the coating on the shape memory, swelling, and release behavior as well as on the mechanical properties of these specimens was thoroughly investigated and the effectiveness of the proposed strategy was demonstrated by the results obtained.

## 1. Introduction

Drug delivery systems (DDSs) intended to be retained within hollow muscular organs may represent an advantageous strategy to maintain effective drug concentrations at the target site for a prolonged period of time, thus potentially improving local treatments and/or bioavailability of different molecules [1,2,3,4,5]. In this respect, intravesical devices have been proposed to reduce the risk of cancer relapse, to fight infections, and to treat other widespread urinary tract diseases. On the other hand, gastro-retentive systems could advantageously be used for treatment of gastric or duodenal ulcers, eradication of *H. pylori*, increased absorption of a range of drugs, and improved efficacy of long-term therapies that are severely impaired by compliance issues.

**Figure 1:**
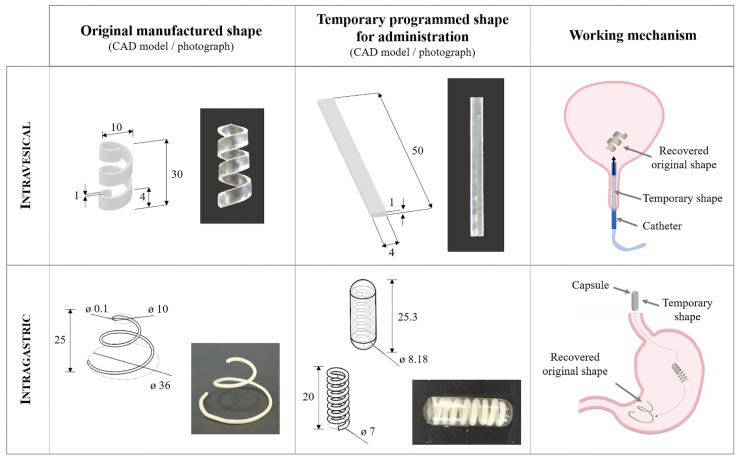
Design concept of expandable DDSs based on SMPs intended for retention in hollow muscular organs (dimensions are in mm).

Depending on the organs involved and the administration mode, different retention mechanisms have been described over the years, such as low-density floatation, high-density sinking, and bioadhesion [6,7,8]. However, when dealing with hollow organs having challenging access through relatively small ducts for the inflow and outflow of biological fluids and containing highly variable amounts of liquids, the strategy based on expansion at the target site was proved especially effective [9]. It entails the use of shapes/sizes that would ensure easy administration of the system, while the subsequent size increase, leading to a bulkier configuration, would make its retention possible. In this respect, swelling and unfolding phenomena have been exploited. The latter may rely on either mechanical/elastic/superelastic deployment or on shape memory, which consists of untethered shape modifications triggered by an external non-mechanical stimulus, such as a change in temperature, moisture, or light [6,10,11,12,13,14,15,16]. Among shape memory polymers (SMPs), pharmaceutical-grade poly(vinyl alcohol) (PVA) has recently been used for the manufacturing of prototypes able to be programmed into space-saving temporary shapes and, upon contact with aqueous media at body temperature, recover the bulky original shapes attained through the production process [17,18]. Due to its well-known safety profile and swelling behavior followed by erosion/dissolution in aqueous media, PVA was selected for the development of DDSs to be retained in the urinary bladder or in the stomach. The ability of such systems to maintain the recovered original shape for a prolonged period of time would depend on their geometry, micro-structure, and composition on one hand, and on the motility of the target muscular organ on the other. Therefore, prototypes having different original shapes were fabricated by hot melt extrusion (HME) and fused deposition modeling (FDM). These techniques were chosen in view of the geometric versatility they would ensure and the possibility of processing materials having diverse properties [19,20,21,22,23,24,25,26,27]. Moreover, 3D printing of a shape memory polymer of pharmaceutical-grade resulted in one of the first applications of 4D printing in pharmaceutics [28,29,30,31,32]. Figure 1 summarizes: (i) original shapes obtained by the manufacturing process, (ii) temporary shapes programmed under appropriate thermo-mechanical protocols, and (iii) possible intravesical as well as intragastric applications of these SMP-based expandable DDSs. The original shapes given under fabrication (e.g., U-, I-, helical-, and S-shape) were designed with a spatial encumbrance suitable for long-lasting residence in the target organ, specifically to avoid early emptying of the DDS and the risk of occlusion of the urethra or the pylorus. On the other hand, the temporary shapes (e.g., I and supercoiled) were conceived for administration through the selected routes (i.e., insertion into the urinary bladder via a catheter or oral intake inside a commercially available hard-gelatin capsule) and achieved by manual deformation. A comprehensive pool of data was obtained from PVA-based samples under various experimental conditions (e.g., volume and type of fluids, temperature, presence of external constraints) [33,34]. Such data were then used to calibrate and validate a mathematical model intended to ease further development of the expandable DDSs allowing for the screening of a broad range of possible compositions and geometries.

However, the relatively short timescale of release from expandable PVA-based DDSs was found to be an issue, especially when dealing with chronic pathologies that would benefit from long-lasting retention at the target site. Indeed, improvement of patient compliance and of treatment efficacy, through reduced dosing frequency and fluctuations in drug plasma concentrations, were indicated as key elements in the development of intra-gastric DDSs intended to treat a range of diseases having great social interest and economic impact, such as acquired immunodeficiency syndrome and hepatitis C [11,13,35,36,37]. For this purpose, an at least 8-h prolonged-release performance would be required from gastro-retentive DDSs [2,6,10]. Interestingly, preliminary attempts to coat the PVA prototypes with low-permeable polymeric films led to promising results [18,34].

Based on these premises and in order to broaden the spectrum of applications of the expandable DDS under development, the aim of the present work was to thoroughly investigate the effects of different permeable insoluble coatings on the mechanical properties, shape memory behavior, and release performance of extruded PVA-based prototypes intended for long-lasting retention within hollow organs. Simple items, having an original I-shape and programmed to take on a temporary U-shape, were employed as screening tools and allopurinol (ALP) was selected as the tracer drug because of its stability even at high temperatures [38].

## 2. Materials and Methods

### 2.1. Materials

PVA (PVA48; Gohsenol^™^ EG 48P, Nippon Gohsei, Tokio, Japan; viscosity of 4% *w/v* solution at 20 °C = 41.3–55.7 (48.5) mm^2^/s); glycerol (GLY; Pharmagel, Milan, Italy); methacrylic acid copolymers, i.e., Eudragit^®^ RL100 and Eudragit^®^ RS100 (Evonik, Darmstadt, Germany); triethyl citrate (TEC; Sigma Aldrich, Milan, Italy); ready-to-use dispersion of methacrylic acid copolymer, i.e., Eudragit^®^ NE (Evonik, Darmstadt, Germany); ethanol (Sigma Aldrich, Milan, Italy); ALP (FarmaQuimica sur S.l., Málaga, Spain; boiling point = 250.36 °C, melting point = 390 °C) [38]; nylon filament containing carbon fibers (Carbonio, TreeD filaments, Milan, Italy).

### 2.2. Methods

#### 2.2.1. Preparation of PVA-Based Formulations

All powders were dried in an oven at 40 °C for 24 h prior to use.

Plasticized polymeric formulation: PVA was placed in a mortar and GLY (15% *w*/*w* calculated on the dry polymer) was added dropwise under continuous mixing.

ALP-containing formulation: ALP was added to the plasticized polymeric formulation (1:9 mass ratio) by mixing in a mortar, immediately before hot-processing.

#### 2.2.2. Hot Melt Extrusion

Prototypes were fabricated by HME using a twin-screw extruder (Haake™ MiniLab II, Thermo Scientific, Milwaukee, WI, USA) equipped with counter-rotating screws and an aluminum homemade die of 1.5 mm in diameter under the following conditions: temperature = 230 °C, screw speed = 25 rpm, maximum torque registered = 250 N·cm. Extruded rods were cut to attain I-shaped samples of 50 mm in length.

#### 2.2.3. Film-Coating

I-shaped samples were coated on their lateral surface (i.e., leaving the two bases of the cylinder uncoated) using an in-house assembled machinery as reported in [34]. Two different coating formulations were employed: (i) an ethanolic solution (final concentration 30% *w*/*v*) containing a Eudragit^®^ RS and Eudragit^®^ RL mixture (3:1 mass ratio) and TEC (15% *w*/*w* calculated on the dry polymeric blend) and (ii) a 30% ready-to-use aqueous suspension of Eudragit^®^ NE. The coating process was performed under the conditions reported in Table 1. Coated samples were withdrawn after 4, 8, and 16 min of processing. After the process, all the coated samples were maintained for 2 h in a ventilated oven at 40 °C.

#### 2.2.4. Characterization

I-shaped samples were evaluated for: weight, coating thickness, thermo-mechanical properties, structure changes upon immersion in aqueous fluids (i.e., swelling profiles), release performance, and shape memory effect. Details on the characterization experiments are reported in the following sections.

##### Weight Gain and Coating Thickness

Uncoated and coated prototypes were checked for mass (n = 6; Gibertini, Milan, Italy). The mass applied per unit area was calculated based on the nominal length (L = 50 mm) and diameter (ø = 1.5 mm) of uncoated cylindrical samples.

**Figure 2:**
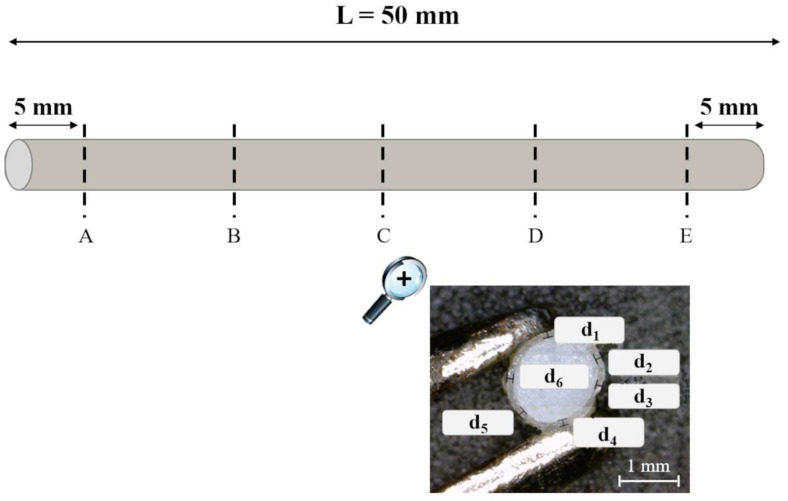
Outline of the A-E positions where I-shaped samples were cut and photographed, showing a cross-section with details relevant to the d_1_-d_6_ coating thickness measurements.

Coated samples (n = 6) were cut at 5 equally spaced positions (A–E) along their length, starting 5 mm away from the ends of the specimen (Figure 2). For each cross-section, the coating thickness was measured at 6 different points (d_1_–d_6_) along the circumference. Photographs of the cross-sections were acquired using a digital microscope (Digital Microscope AM-413T, Dino-Lite, Milan, Italy; resolution = 1.3 Megapixel − 1280 × 1024) and processed by an image-analysis software (ImageJ, Milan, Italy).

##### Thermal Properties

Differential scanning calorimetry (DSC) analyses were performed by means of a DSC Q100 calorimeter (TA Instruments, New Castle, DE, USA), using nitrogen as a purge gas (50 mL/min). Samples of about 10 mg were cut from uncoated and coated specimens. Eudragit^®^ RS/RL and Eudragit^®^ NE films prepared by casting (i.e., by pouring liquid formulations into circular silicon molds and drying in a ventilated oven for 12 h at 40 °C) were also analyzed. Samples were heated at 10 °C/min from −50 °C to 160–240 °C (the heating ramp never exceeded 180 °C in the case of specimens coated with the Eudragit^®^ RS/RL formulation, because the coating was prone to degradation at higher temperatures).

##### Mechanical Properties

Mechanical properties were studied under tensile and compression conditions at room temperature, by means of an electromechanical dynamometer (Instron 3366, Norwood, MA, USA). The tests were carried out before and after immersion of the samples (n = 3) in distilled water at 20 °C for increasing times (up to 24 h).

The mechanical properties in the specimen length direction (i.e., the longitudinal properties) were evaluated through uniaxial tension tests performed with a crosshead speed of 10 mm/min on I-shaped samples having a gauge length of 20 mm. Two different load cells (i.e., 500 N and 50 N) were employed for dry and swollen prototypes, respectively, according to their different stiffness.

The results were expressed in terms of nominal stress (σ) versus strain (ε) curves, with σ and ε calculated as follows:

(1) $$\sigma =\frac{4P}{{\pi d}^{2}}$$

(2) $$\varepsilon =\frac{u}{l_{t}}$$

where P is the measured load, d is the mean diameter of the sample, u is the crosshead displacement, and l_t_ is the gauge length.

From σ versus ε curves, the tensile modulus (E), taken as the initial slope of the curves, and the stress at 10% strain (σ_10%_) were evaluated.

The mechanical properties in the specimen radial direction (i.e., the transverse properties) were evaluated through compression tests. The experiment was performed by laying the I-shaped sample on its long side between compression plates and applying the load along the diameter with a crosshead speed of 0.5 mm/min. Two different load cells (i.e., 10 kN and 500 N) were employed, depending on the specimen stiffness.

The results were expressed as normalized load (P_N_) versus normalized displacement (u_N_) curves, with P_N_ and u_N_ calculated as follows:

(3) $$P_{N}=\frac{P}{l_{c}d}$$

(4) $$u_{N}=\frac{u}{d}$$

where P is the measured load, d is the mean diameter of the sample, l_c_ is the mean length, and u is the crosshead displacement.

From P_N_ versus u_N_ curves, the transverse stiffness parameter (S*) was calculated as the ratio of normalized load and normalized displacement for u_N_ = 0.1:

(5) $$S^{*}=\frac{P_{N}\left(u_{N}=0.1\right)}{0.1}$$

##### Swelling

Coated and uncoated samples (n ≥ 4) were immersed in unstirred distilled water at room temperature and collected after different immersion times (t_imm_) to evaluate the relevant water uptake and physical as well as mechanical changes upon swelling. Based on the peculiar hydration, swelling, and dissolution properties of the samples, different t_imm_ were considered, ranging from 10 min to 24 h. In particular:

₋ t_imm_ for uncoated samples = 10 min, 30 min, 1 h, 2 h, 4 h, and 6 h;
₋ t_imm_ for samples coated with Eudragit^®^ RS/RL = 10 min, 30 min, 1 h, 4 h, and 6 h;
₋ t_imm_ for samples coated with Eudragit^®^ NE = 10 min, 30 min, 1 h, 4 h, 6 h, 10 h, and 24 h.

After withdrawal, each sample was gently blotted. Initial mass (m_i_) and mass after immersion in water (m_w_) were measured (analytical balance, Gibertini, Milan, Italy) to calculate the water uptake as mass variation percentage (Δm):

(6) $$\Delta m\;\left(\%\right)=\frac{m_{w}-m_{i}}{m_{i}}\times 100$$

Each sample was placed flat under a microscope (Leica MS5, Leica, Munich, Germany), and the relevant diameter was measured by means of the microscope micrometer at the B, C, and D positions as shown in Figure 2. The mean diameter was evaluated before and after immersion in water ($\bar{d_{i}}$ and $\bar{d_{w}}$, respectively). The diameter percentage variation (Δd) was calculated as follows:

(7) $$\Delta d\;\left(\%\right)=\frac{\bar{d_{w}}-\bar{d_{i}}}{\bar{d_{i}}}\times 100$$

**Figure 3:**
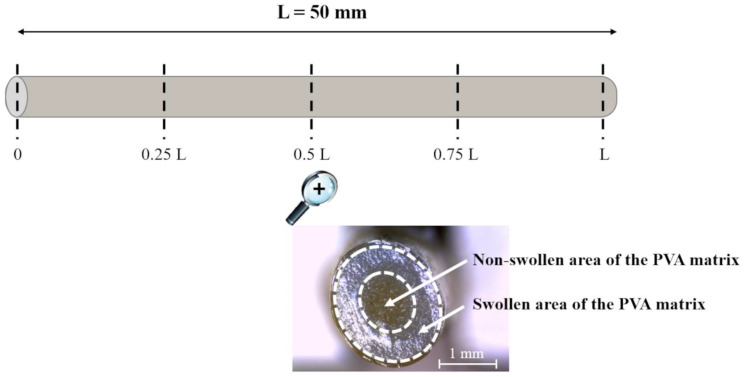
Outline of the different positions where the swollen I-shaped samples were cut and photograph of a cross-section with details relevant to the different areas measured.

Hydrated/swollen samples were cut into 4 equal segments, so that their cross-sections could be observed and photographed under the microscope (Leica DMS300, Leica, Munich, Germany) at 5 equally spaced positions along their longitudinal axis, i.e., the two uncoated surfaces (0 and L positions) and the 3 internal positions expressed as fractions of the total length (L) of the specimen (0.25L, 0.5L, and 0.75L, respectively) (Figure 3). The typical appearance of a cross-section is also reported in Figure 3, showing a clearly distinguished swollen area (translucent part of the cross-section) and a non-swollen area (opaque), separated by dashed lines. The circumference of the non-swollen area highlighted in Figure 3, separating the rubbery region that has already interacted with water from the merely glassy portion that has not yet hydrated, corresponds to the swelling front. The area of the swollen and the non-swollen portions of the PVA matrix (excluding the coating layer in the coated samples) was measured by means of the above-mentioned image analysis software. For each t_imm_ considered, a different specimen was employed.

The swollen area was calculated as a percentage of the total matrix area as follows:

(8) $$\mathrm{Swollen}\;\mathrm{PVA}\;\mathrm{area}\;\left(\%\right)=\frac{\mathrm{Swollen}\;\mathrm{PVA}\;\mathrm{area}}{\mathrm{Swollen}\;\mathrm{PVA}\;\mathrm{area}\;+\;\mathrm{Non}-\mathrm{swollen}\;\mathrm{PVA}\;\mathrm{area}}\times 100$$

##### Release

Coated and uncoated prototypes were tested for release using a USP38 dissolution apparatus 2 (paddle speed = 50 rpm, 900 mL of HCl 0.1 N kept at 37 ± 0.5 °C; Distek, North Brunswick Township, NJ, USA; n = 6), connected to a pump (IPC Ismatec™, Thermo Fisher Scientific, Milan, Italy) for the automatic collection of fluid samples and to a spectrophotometer for assay (Lambda 35, Perkin Elmer, Milan, Italy; 1 mm cuvette path length, 251 nm λ_max_). The amount of ALP released at each time point was determined from a calibration curve (y = 5.6258x + 0.0022, R^2^ = 0.9999).

##### Shape Memory Effect

Coated and uncoated prototypes were programmed to take on a temporary U-shape, according to a previously developed method [17]. Special templates fabricated by FDM 3D printing were employed for programming the desired temporary shape. Computer-aided design (CAD) files were purposely created using Autodesk^®^ Inventor^®^ Professional 2019 software version 14.0 (Autodesk Inc., Milan, Italy). The files were saved in .stl format and imported to the printer software (Simplify 3D, Milan, Italy). Next, 3D printing was performed by a Kloner3D 240^®^ Twin (Kloner3D, Florence, Italy) printer equipped with 0.5 mm nozzle (printing temperature = 230 °C, infill = 100%, layer height = 0.10 mm, printing speed = 50 mm/s). A commercially available nylon filament containing carbon fibers was used as received. Coated and uncoated prototypes were heated up to 55 °C (i.e., above the glass transition region) and manually deformed with the aid of the specially printed templates. The temporary shape was then fixed by cooling the specimens inside the templates at −20 °C for at least 4 h before testing.

Recovery of the original shape was studied upon immersion of the deformed samples into a crystallization vessel containing 250 mL of HCl 0.1 N, kept at 37 ± 0.5 °C by means of a thermoregulated bath. Changes in shape were monitored using a digital camera (GoPro Hero Session, San Mateo, CA, USA; n = 3) positioned 13 cm above the specimens. The photographs acquired were processed using the previously mentioned image analysis software, and the recovery index (RI) was calculated as follows:

(9) $$\mathrm{RI}\;\left(\%\right)=\frac{\alpha -\alpha_{p}}{\pi -\alpha_{p}}\times 100$$

where α is the angle measured between the two arms of the U-shaped sample and α_p_ is the angle obtained in the programming phase (expressed in rad). RI was represented as a function of time [17].

## 3. Results

In order to prolong release from the expandable SMP-based retentive prototypes under development, a strategy involving (i) HME of a PVA characterized by higher molecular weight with respect to the previously tested ones, and (ii) coating of the extruded devices with insoluble, low-permeable films was pursued. In this respect, the commercially-available pharmaceutical-grade of PVA having the highest molecular weight (PVA48) was selected as the main component of the matrix systems, and an ethanolic solution of Eudragit^®^ RS/RL (3:1 mass ratio) or a ready-to-use aqueous suspension of Eudragit^®^ NE was applied to the PVA-based samples to achieve coatings of different permeability and thickness [39,40].

### 3.1. Coating of the Expandable Prototypes

An in-house assembled equipment was employed to coat I-shaped extruded prototypes [34]. This was purposely conceived for specimens with an asymmetrical shape, which represented unusual substrates with respect to solid cores traditionally undergoing film-coating (e.g., tablets, capsules). The equipment consisted of a spray unit and a rotating device carrying the samples, thus allowing them to be fully coated along their length while both ends (i.e., the top and bottom bases of the cylindrical matrix) were left uncoated. The coating process was performed for 4, 8, and 16 min. In Table 2, all coated samples are listed along with the relevant manufacturing details and identification codes.

**Figure 4:**
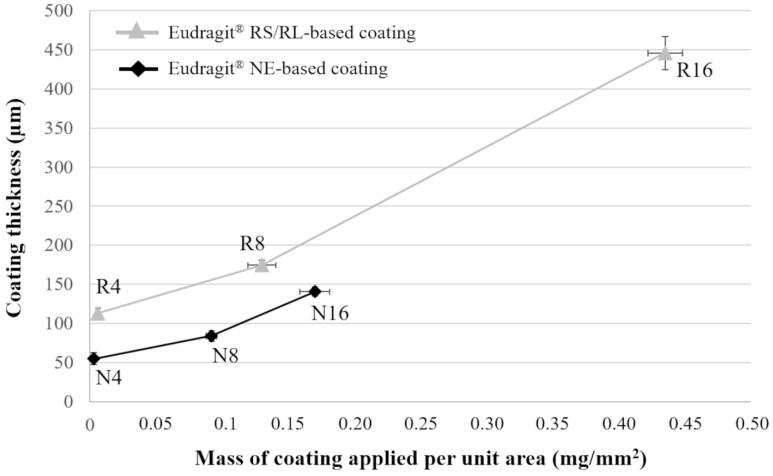
Coating thickness versus amount of coating applied to I-shaped samples.

As expected, longer process times led to increasing coating thicknesses (Table 3). Notably, the greatest variability in thickness (CV in the 12–16 range) was found for N4 samples having a < 50 μm thick Eudragit^®^ NE coating. CV values relevant to all the other coated prototypes were much lower, indicating that a reproducible process was set up. The growth trend of thickness versus mass per unit area of the coating applied was quite similar for the two formulations considered (Figure 4). However, the thickness achieved in the same process time turned out lower when dealing with the Eudragit^®^ NE aqueous suspension. Indeed, as water required longer evaporation times, the spray rate of the Eudragit^®^ NE formulation needed to be decreased to avoid swelling/dissolution of the underlying PVA matrix.

### 3.2. Thermal Analysis

**Figure 5:**
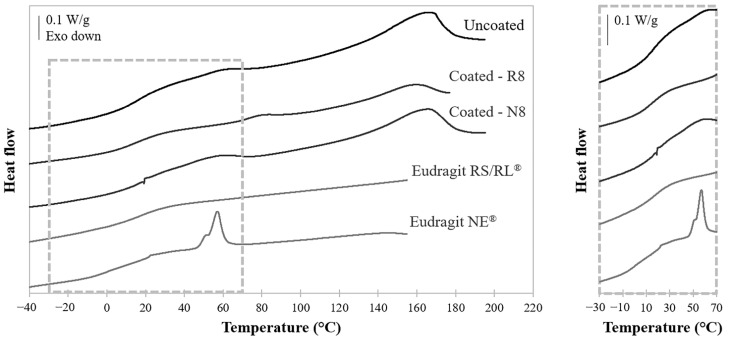
Thermograms of uncoated and coated (i.e., R8 and N8) samples, and of free Eudragit^®^ RS/RL and Eudragit^®^ NE films; the insert is a magnification of the glass transition regions.

The glass transition region of the materials in use, which has a fundamental role in the activation of the shape memory effect, was evaluated by DSC. In order to assess any possible influence of the coating on the overall glass transition of PVA, tests were performed on uncoated prototypes, on free Eudragit^®^ RS/RL and Eudragit^®^ NE films, and on coated systems. In particular, the glass transition region was identified as an inflection in the heating traces. Uncoated PVA-based samples and free Eudragit^®^ films displayed a broadly distributed glass transition region, approximately between 0 and 30 °C, corresponding to the smooth inflections highlighted in the insert of Figure 5. In addition, the trace of uncoated samples pointed out an endothermic peak starting at approximately 160 °C due to PVA melting, while that of the Eudragit^®^ NE film showed a characteristic endothermic signal slightly below 60 °C, which could be ascribed to the melting of the emulsifier contained in the commercial coating suspension, i.e., nonoxynol [41].

R8 and N8 thermograms were analogous to those of the relevant components, featuring (i) a glass transition signal to be ascribed to moisture evaporation and (ii) the PVA melting peak at higher temperatures. This result confirmed that the coating process did not bring about alterations of the thermal behavior of the underlying formulation. Moreover, such findings suggested that the presence of the coatings should have a minor impact on the shape recovery process of the final systems. In fact, shape recovery was intended to occur at body temperature, i.e., above the glass transition region of both components.

### 3.3. Release Tests

**Figure 6:**
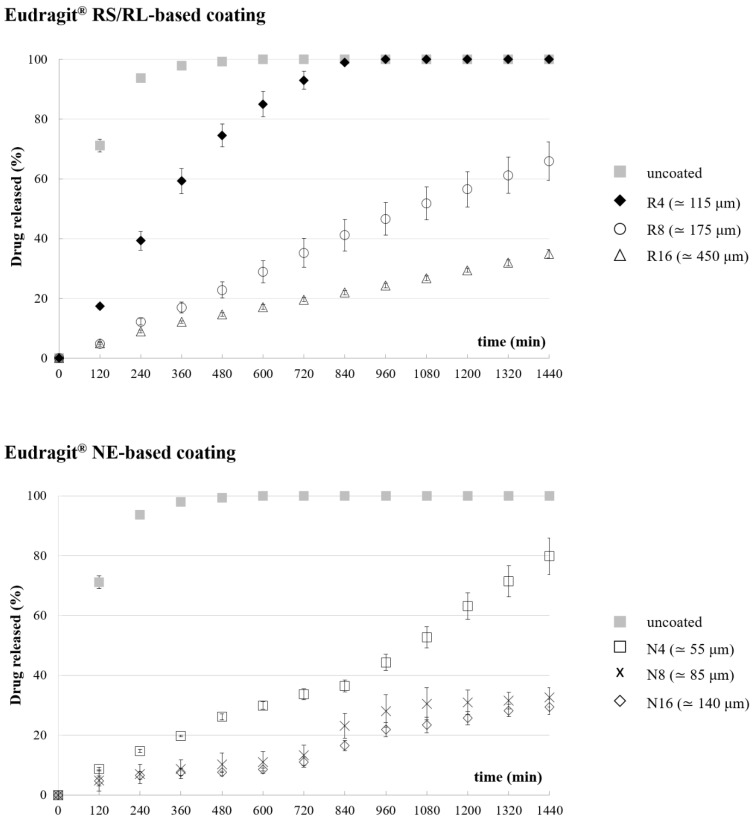
Average release profiles from uncoated and coated samples.

According to the soluble swellable nature of the PVA matrix, drug release from uncoated prototypes was prolonged over 6 h. Regardless of the formulation employed and of the coating thickness, all coated prototypes showed longer release profiles than uncoated ones (Figure 6), even exceeding 48 h in some cases. Indeed, the insoluble films applied to the lateral surface of the cylindrical PVA core would reduce the rate of radial penetration of fluid and that of outward diffusion of the drug, possibly preceded by a lag phase. However, the coated samples were also characterized by a very limited uncoated surface area (about 1.5% of the total) immediately available for interaction with the aqueous medium—that relevant to the film-free cylinder bases. Dissolution of drug particles from these uncoated surfaces, occurring before the formation of a gel barrier, could thus counteract the lag phase.

As the uncoated prototypes were shown to swell during the release test, the coated specimens were carefully observed to highlight any modifications in shape and/or dimensions of the PVA core and possible impact on the coating integrity. Penetration of the aqueous medium was expected to start from the uncoated surfaces proceeding lengthwise, and to also occur through the coated lateral surface moving towards the center of the matrix. In this regard, the rate of radial penetration should be affected by the formulation and thickness of the coating. In the Eudragit^®^ RS/RL-coated samples, the applied film was apparently able to adapt to the expansion of the PVA matrix in the radial direction until a breaking point was reached. This impacted on drug release, which was faster from R4 samples having ~115 μm thick coating. Considering R8 prototypes with a ~175 μm coating thickness, a remarkable slowdown in release was observed. Conversely, a further increase in the coating thickness up to ~450 μm (i.e., R16 samples) resulted in a much smaller reduction in the release rate.

Eudragit^®^ NE films were shown more efficient in reducing the release rate, in accordance with their known poor permeability [42,43]. Moreover, the Eudragit^®^ NE coating acted as a stronger physical constraint to the PVA core swelling and maintained its integrity over time. Such a behavior, which is well described in the literature as the swelling-restriction mechanism, affected the release kinetics while promoting the longitudinal squeeze-out of the swollen polymeric matrix from the uncoated bases of the I-shaped samples [44,45,46]. This was also highlighted by a change in the slope of the release curves. Specimens coated with the lowest amount of Eudragit^®^ NE (i.e., N4) already exhibited a reduction in the release rate, while increasing the film thickness from ~80 μm to ~140 μm led to a non-proportional decrease in such a parameter. Indeed, the overall release profiles from N8 and N16 coated samples turned out to be comparable. This could indicate the presence of a threshold value in the coating thickness, beyond which penetration of water through the film and outward release of the tracer drug would in any case be effectively slowed down. Therefore, at this stage, the main release mechanism would be the diffusion of the drug through the swollen uncoated bases of the cylindrical core, which is in fact independent of the lateral coating layer. Based on the results obtained, the following characterizations were performed on the N4 and N8 samples only.

### 3.4. Swelling Behavior

**Figure 7:**
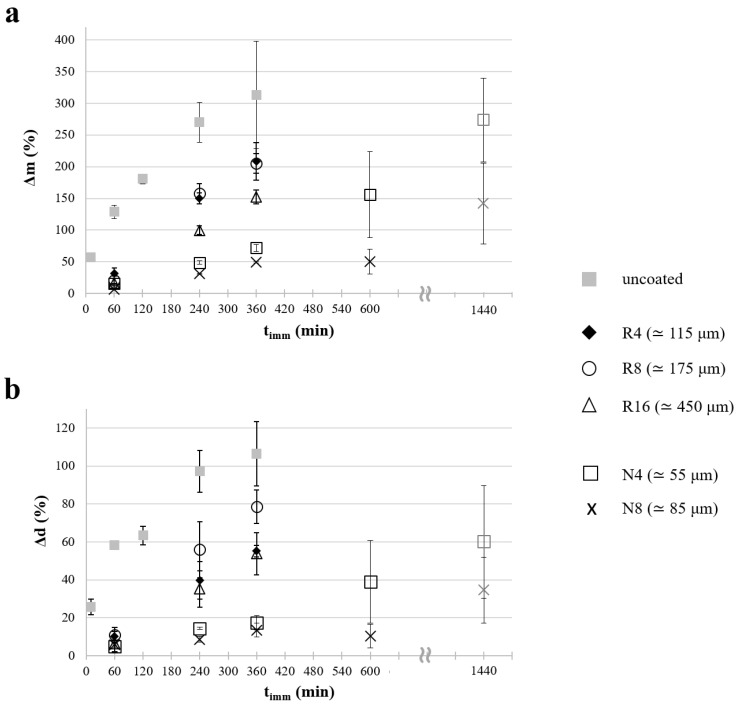
Profiles of (**a**) mass variation and (**b**) diameter increase plotted versus immersion time relevant to uncoated and coated samples.

The study of mass variation (Δm) and diameter increase (Δd) of samples as a function of the relevant immersion time in water, under room temperature and unstirred conditions, provided further insight into the swelling behavior of uncoated and coated specimens (Figure 7a and Figure 7b, respectively). This experimental setup was chosen as it allowed a slower interaction with aqueous fluids, thus highlighting possible differences in the behavior of the samples.

As expected, all prototypes showed an increase in water uptake for longer t_imm_, with the uncoated samples swelling relatively faster than the coated ones. The results obtained were generally consistent with the overall release data previously discussed and the morphological changes observed. The coating, regardless of its composition, led to a decrease in the rate of aqueous fluid penetration, both by acting as a barrier to fluid diffusion and/or by mechanically hindering the expansion of the PVA matrix core (i.e., swelling restriction mechanism) [46]. After 1h immersion, a 130% water uptake was reached by the uncoated specimens, whereas for the coated ones Δm was approximately in the 10–30% range, depending on the type and thickness of the coating. As t_imm_ increased, the influence exerted by the coating layer became more evident.

Given the relatively slow mass variation pointed out by samples coated with Eudragit^®^ NE, the experiments lasted up to 24 h, which proved sufficient to achieve weight gains similar to those attained with the other specimens under investigation.

A consistent behavior was observed in terms of mean diameter increase, as highlighted in Figure 7b. Indeed, as a consequence of water uptake, the sample diameter tended to increase, with greater Δd values for longer immersion times, thinner coatings, and more permeable coating formulations.

Notably, Δd was especially small for specimens coated with Eudragit^®^ NE, even in the case of N4 samples, probably due to the relatively low permeability of the coating films and the mechanical constraint they exerted on the PVA swelling. This was particularly evident for long immersion times.

**Figure 8:**
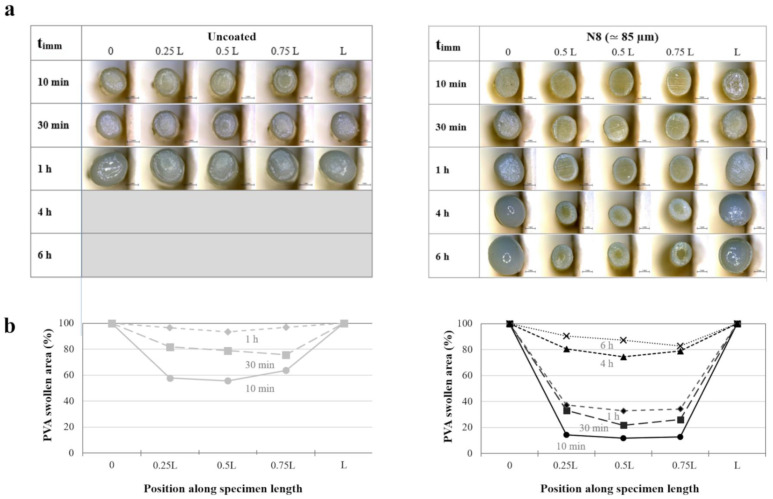
(**a**) Photographs of cross-sections of uncoated and coated N8 prototypes at different positions along their length after increasing immersion times and (**b**) profiles of the corresponding swollen area.

Due to the peculiar configuration of the samples (i.e., presence of uncoated ends), aqueous fluid penetration was supposed to proceed at different rates along their longitudinal and radial axes, which might impact on the overall swelling behavior of the PVA-based core. Therefore, it was interesting to observe different cross-sections of uncoated and coated prototypes (i.e., surfaces cut at diverse positions along the specimen length) at increasing t_imm_. The selected positions were the uncoated ends and 3 equally spaced inner points (i.e., 0 and L versus 0.25L, 0.5L, 0.75L, as indicated in Figure 3). By way of example, Figure 8 shows photographs of the cross-sections of an uncoated specimen and a coated N8 one at increasing t_imm_, together with profiles describing the swollen PVA area along the longitudinal axis of the samples.

**Figure 9:**
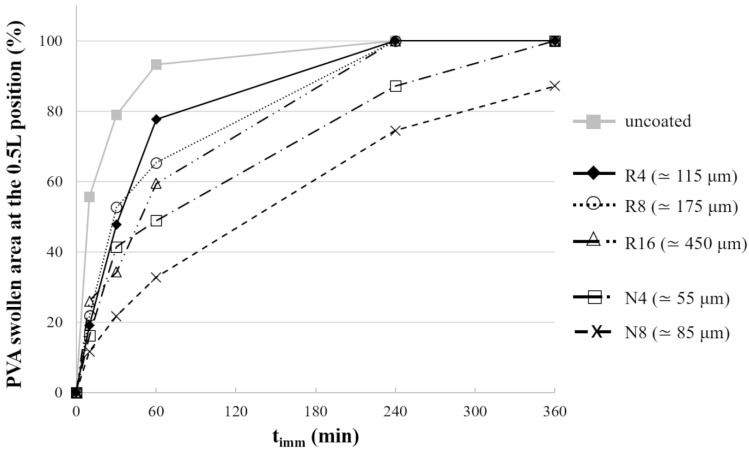
Profiles of swollen area in the central cross-section of uncoated and coated samples as a function of the immersion time.

Overall, the images suggested the presence of a swelling front in the PVA matrix moving in the radial direction as a function of t_imm_. Notably, for both uncoated and coated specimens, the ends were rapidly fully swollen. Conversely, at the 0.25 L, 0.5 L, and 0.75 L positions, the polymer was only partially swollen, with the swollen area increasing at different rates for uncoated and coated samples. To better highlight the effect of the various coatings, the percentage swollen area in the cross-section corresponding to the center of the specimens (i.e., 0.5L) was considered (Figure 9). This representation was helpful to confirm that the time required for water to penetrate through the entire cross-section depended on the type and thickness of the applied coating. Indeed, by comparing samples coated with the same formulation, it was evident that a thicker coating would result in a reduced swelling rate.

### 3.5. Mechanical Tests

During release tests, the samples showed macroscopic changes and increased flexibility upon interaction with aqueous fluids. Thus, their mechanical properties seemed worthy of further study, especially because the above-mentioned modifications might impact the retentive performance of the expandable system under development and its safe use.

Mechanical tests were performed at room temperature, under tensile and compression conditions, and the results obtained were expressed as a function of t_imm_. While tensile tests were carried out along the sample axis, compression tests involved applying a force perpendicularly to the longitudinal axis of the samples. In this respect, the combination of tensile (i.e., axial) and compression (i.e., transverse) experiments could provide comprehensive information in light of the intrinsically anisotropic structure of the specimens under investigation.

**Figure 10:**
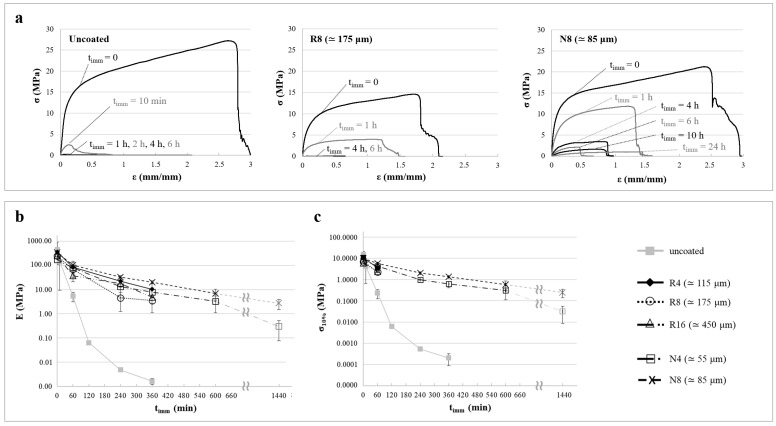
(**a**) Stress versus strain curves relevant to uncoated, R8, and N8 specimens tested under tensile conditions after different immersion times, and profiles of (**b**) E as well as of (**c**) σ_10%_ as a function of immersion time.

Tensile test results relevant to uncoated, R8, and N8 samples are shown in Figure 10a as stress versus strain curves. Two different parameters were calculated: the tensile modulus (E) and the stress corresponding to a strain equal to 10% (σ_10%_), which are reported in Figure 10b and c, respectively. While E represented the stiffness of the specimens in the longitudinal direction, σ_10%_ was used to compare the stress level at a given strain for different samples, as it was not possible to measure the ultimate tensile strength. Indeed, the load drops highlighted in Figure 10a did not correspond to specimen failure, which was never observed, but to its slippage from the clamps. However, in Figure 10c, σ_10%_ data for R4, R8, and R16 were not reported because, since coating failure (i.e., localized cracking along the circumference) was observed at strain lower than 10%, they were not considered useful for comparison with intact samples.

The uncoated samples underwent a drastic drop in the stress values within 1 h of immersion in water. For this reason, stress versus strain curves of specimens with t_imm_ ≥ 1 h are barely visible in Figure 10a, while both E and σ_10%_ decreased by 5–6 orders of magnitude in 6 h. Indeed, the former parameter moved from hundred MPa to approximately 1 kPa, while the latter dropped from just over 10 MPa to fractions of kPa. By contrast, coated specimens immersed in aqueous fluid for similar times exhibited a less marked reduction in these properties. Both Eudragit^®^ RS/RL and Eudragit^®^ NE coatings, as long as their integrity was maintained, limited the decrease in mechanical properties (about 1–2 orders of magnitude in 6 h, with a retention of about 10 MPa in E and about 1 MPa in σ_10%_). This happened because the coating layer generally hindered the swelling of the PVA matrix underneath and maintained its stiffness, while supporting most of the longitudinal load. This latter aspect may also explain why varying the coating thickness had only minor consequences on tensile properties, although the Eudragit^®^ NE coating limited swelling of the PVA core much more than the Eudragit^®^ RS/RL one did.

**Figure 11:**
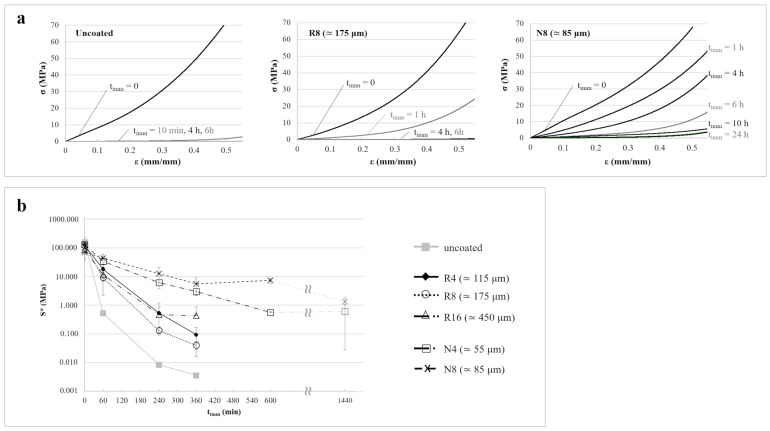
(**a**) Normalized load versus normalized displacement curves relevant to uncoated, R8, and N8 specimens tested under compressive conditions after different immersion times and (**b**) profiles of S* as a function of immersion time.

For comparison purposes, the compression behavior of the same samples previously described is presented in Figure 11a, in which normalized load versus normalized displacement curves are shown. Notably, the compression configuration could not be exactly associated with a simple deformation state. Indeed, while in tensile condition, both the PVA core and the external coating were subjected to the same elongation; in the compression experiments, the strain and the applied stress changed from point to point along the load direction due to the cylindrical shape of the specimen. The curves displayed a slope that gradually increased as the upper compression plate came in contact with progressively wider sections of the specimen during descent. The qualitative evolution of the response for increasing t_imm_ was similar to that of tensile stress versus strain curves. In fact, 1 h in water was enough to cause a drastic drop in normalized load values for the uncoated samples, while the coated ones sustained higher stresses, especially when dealing with those coated with Eudragit^®^ NE. This could only be attributed to the different amount of water absorbed.

A stiffness parameter (S*), evaluated at a normalized displacement equal to 0.1, was introduced to better describe the mechanical properties in the transverse direction of compressed specimens (Figure 11b). The effect of t_imm_ and of the type and thickness of the coating on S* was comparable to that observed for E during tensile tests, although differences between the various coatings became more evident. For instance, after 6 h in water, the decrease in stiffness was around 5 orders of magnitude for uncoated specimens, around 3 for specimens coated with Eudragit^®^ RS/RL, and less than 2 for Eudragit^®^ NE-coated ones. In the latter case, the decrement did not exceed 2 orders of magnitude, even after 24 h of immersion. Focusing on the same coating composition, S* were higher with increasing thicknesses. These results suggested that the mechanical properties in the transverse direction would more closely be related to the degree of swelling of the PVA core, which—for a given immersion time—was higher in prototypes having more permeable or less thick coatings. Indeed, the compliance of the swollen core played a major role in the overall compliance of the specimen, whereas the contribution of the coating stiffness was limited, in contrast to what observed in the longitudinal direction.

### 3.6. Shape Memory Effect

Shape memory tests were carried out to verify whether the presence of the coating, shown to play a key role in defining the swelling, release, and mechanical performance of the prototypes, could impact the retention mechanism of the expandable device under development. In detail, the experiments were carried out by evaluating the ability of coated samples to recover their original I-shape after deformation and fixing into a temporary U-shape.

**Figure 12:**
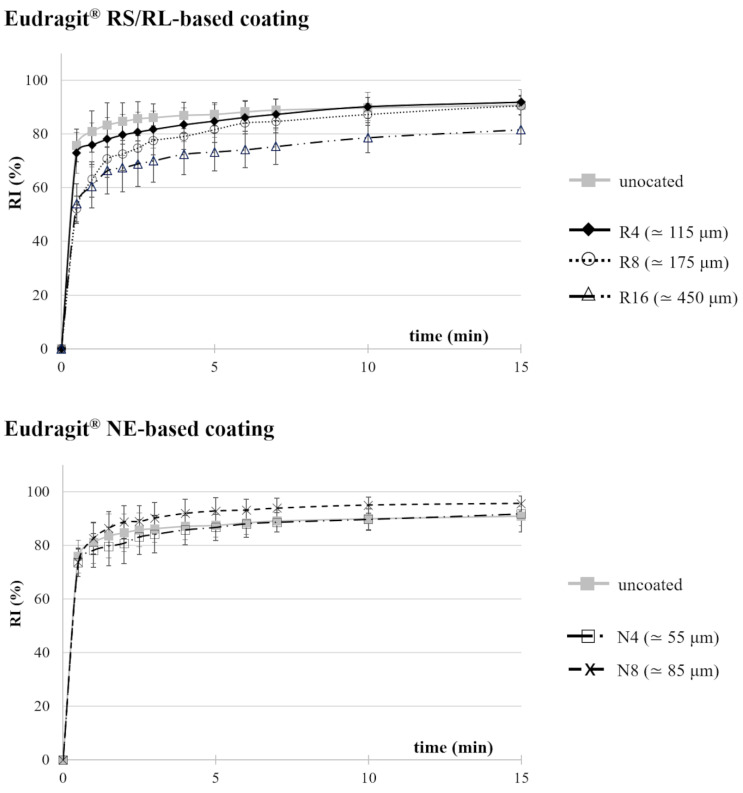
RI versus time curves relevant to I-shaped samples coated with different formulations.

Recovery index (RI) versus time curves relevant to both uncoated and coated specimens are reported in Figure 12. The focus was on the first 15 min of testing, during which most of the recovery process occurred. All the prototypes were able to recover their original I-shape within 1 h of immersion, reaching RI values greater than 70% in the first 5 min of testing.

The overall shape memory behavior was similar for uncoated and coated samples: all of them recovered more than 50% of their original shape in the first 30 s, even exceeding 70% in the case of Eudragit^®^ NE-coated ones. As the coating was expected to reduce the rate of water penetration into the PVA matrix underneath, such a result suggested that the recovery process would mainly be thermally driven, being particularly fast as the working temperature was well above the material glass transition region [33].

For R8 and R16 samples, the recovery process was slightly slower (RI between 65% and 80% after 5 min). Indeed, R16 specimens never recovered as much as the others in the initial 15 min of testing, which was ascribed to a hindering effect exerted by the Eudragit^®^ RS/RL coating that, at the investigated thickness values, would limit the expansion of the PVA matrix. By contrast, recovery of the original shape in the case of Eudragit^®^ NE-coated samples seemed faster, and the relevant rate increased from 55 to 85 µm coating thicknesses. It could be hypothesized that the flexibility of the film, which turned out to be much higher than that of the Eudragit^®^ RS/RL one, might have a positive impact during these experiments, making the film act as a sort of rubbery envelope, helping the shape recovery process. In support of Eudragit^®^ NE flexibility, it is useful to be reminded of the following: it is a neutral ester polymer with no hydrogen bonds or other intermolecular forces; its glass transition temperature is approximately 5 °C, whereas that of Eudragit^®^ RS/RL is of about 17 °C; it has a large strain at break (static strain > 600%) at room temperature, so that its dispersion has a low minimum film formation temperature and does not require the use of plasticizers in traditional coating [47,48].

## 4. Conclusions

Expandable DDSs manufactured by HME and FDM based on shape memory PVA of pharmaceutical-grade were proposed as a viable platform for targeting the delivery of active ingredients into hollow muscular organs. Among SMPs already employed in 4D printing of DDSs, this is the first pharmaceutical-grade polymer used as such and not chemically modified, which would require a costly and time-consuming safety evaluation for implementing it within drug product manufacturing. In this work, the application of coatings having different permeability was demonstrated to be an effective strategy to limit the interaction with aqueous fluids of the underlying PVA matrix and to reduce the outward diffusion of the drug conveyed, thus extending drug release. This would in principle broaden the spectrum of therapeutic applications of the expandable DDSs proposed.

Hot melt extruded prototypes of simple shape were employed, which were deemed suitable for setting up cost-effective screening protocols for a preliminary study of mechanical properties and swelling behavior. They were manufactured starting from a high-molecular weight PVA, loaded with a tracer, and film-coated with different amounts of Eudragit^®^ RS/RL or Eudragit^®^ NE. All the swelling experiments, regardless of whether water uptake, dimensional change, or local progression of the swelling front was considered, highlighted the possibility of modulating the interaction of PVA prototypes with aqueous fluids by acting on the thickness and composition of the applied coating. The presence of different coatings was also shown to affect the evolution of the mechanical properties of samples after the relevant immersion in aqueous medium for increasing periods of time. In detail, Eudragit^®^ NE coatings exhibited a greater ability to modulate water penetration and to limit the dimensional changes of the PVA core, prolonging drug release far beyond 24 h. Although characterized by lower thicknesses with respect to Eudragit^®^ RS/RL coated samples, they were also proved particularly effective in promoting the ability of the prototype to maintain adequate mechanical strength upon immersion in aqueous media. Interestingly, the efficiency of Eudragit^®^ NE coatings would allow a high degree of release control to be attained, even at relatively low thicknesses.

The drug release profiles and the mechanical behavior observed, coupled with the ability of the coated samples to maintain the desired shape memory effect responsible for their retention in hollow organs, were considered as particularly promising results. In this respect, the feasibility of different manufacturing techniques, ranging from HME to 3D printing and coating, to fine-tune the performance of the DDS intended for organ retention resulted a proof of concept for further research towards application of 4D printing in the pharmaceutical field.

## Figures and Tables

**Table 1:** Coating process conditions.

Coating Formulation	Spray Rate (mL/min)	Nebulized Air Pressure (bar)	Pattern Pressure (bar)	Drying Air Temperature (°C)	Drying Air Flow (m^3^/h)	Sample Rotation Speed (rpm)
Eudragit^®^ RS/RL ethanolic solution	7	0.75	1	40	50	2.3
Eudragit^®^ NE aqueous suspension	2.1	0.5	0.75	60	65	1.5

**Table 2:** Uncoated and coated samples with the relevant manufacturing details and identification codes.

	Coating Formulation	Coating Process Time (min)	Code
PVA-based samples 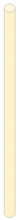	None	0	uncoated
	Eudragit^®^ RS/RL-based coating 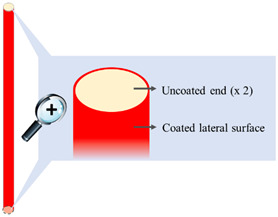	4	R4
		8	R8
		16	R16
	Eudragit^®^ NE-based coating 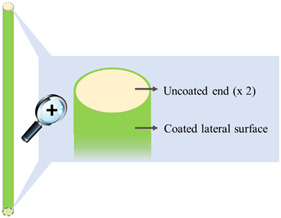	4	N4
		8	N8
		16	N16

**Table 3:** Thickness of (a) Eudragit^®^ RS/RL and (b) Eudragit^®^ NE-based coatings applied to I-shaped samples. The coefficient of variation (CV) is reported in brackets.

(a)	Code	Thickness, µm (CV)				
		Position				
		1	2	3	4	5
	R4	107.8 (4.7)	110.1 (5.6)	113.6 (5.7)	117.2 (5.4)	116.7 (6.4)
	R8	173.5 (2.1)	171.4 (2.1)	175.2 (1.3)	178.6 (4.3)	174.3 (4.9)
	R16	443.3 (6.8)	455.8 (1.4)	449.0 (4.1)	450.8 (4.9)	428.5 (3.8)

**(b)**	**Code**	**Thickness, µm (CV)**				
		**Position**				
		**1**	**2**	**3**	**4**	**5**
	N4	55.2 (16.3)	56.8 (12.3)	56.1 (12.0)	54.2 (15.6)	53.6 (14.8)
	N8	75.6 (5.3)	86.5 (7.9)	86.1 (7.7)	83.9 (4.1)	88.1 (8.0)
	N16	132.7 (4.6)	140.0 (1.6)	144.4 (3.7)	143.2 (2.9)	142.6 (3.2)

## Data Availability

Data are available on request.

## References

[1] Bardonnet P., Faivre V., Pugh W., Piffaretti J., Falson F. (2006). Gastroretentive dosage forms: Overview and special case of *Helicobacter pylori*. J. Control. Release.

[2] Gupta R., Tripathi P., Bhardwaj P., Maho A. (2018). Recent advances in gastro retentive drug delivery systems and its application on treatment of *H. Pylori* infections. J. Anal. Pharm. Res..

[3] Lopes C.M., Bettencourt C., Rossi A., Buttini F., Barata P. (2016). Overview on gastroretentive drug delivery systems for improving drug bioavailability. Int. J. Pharm..

[4] Melocchi A., Uboldi M., Cerea M., Foppoli A., Maroni A., Moutaharrik S., Palugan L., Zema L., Gazzaniga A. (2021). Shape memory materials and 4D printing in pharmaceutics. Adv. Drug Deliv. Rev..

[5] Vrettos N.-N., Roberts C.J., Zhu Z. (2021). Gastroretentive Technologies in Tandem with Controlled-Release Strategies: A Potent Answer to Oral Drug Bioavailability and Patient Compliance Implications. Pharmaceutics.

[6] Klausner E.A., Lavy E., Friedman M., Hoffman A. (2003). Expandable gastroretentive dosage forms. J. Control. Release.

[7] Kumar M., Kaushik D. (2018). An overview on various approaches and recent patents on gastroretentive drug delivery systems. Recent Pat. Drug Deliv. Formul..

[8] Palugan L., Cerea M., Cirilli M., Moutaharrik S., Maroni A., Zema L., Melocchi A., Uboldi M., Filippin I., Foppoli A. (2021). Intravesical drug delivery approaches for improved therapy of urinary bladder diseases. Int. J. Pharm. X.

[9] Maroni A., Melocchi A., Zema L., Foppoli A., Gazzaniga A. (2020). Retentive drug delivery systems based on shape memory materials. J. Appl. Polym. Sci..

[10] Altreuter D.H., Kirtane A.R., Grant T., Kruger C., Traverso G., Bellinger A.M. (2018). Changing the pill: Developments toward the promise of an ultra-long-acting gastroretentive dosage form. Expert Opin. Drug Deliv..

[11] Bellinger A.M., Jafari M., Grant T.M., Zhang S., Slater H.C., Wenger E.A., Mo S., Lee Y.-A.L., Mazdiyasni H., Kogan L. (2016). Oral, ultra–long-lasting drug delivery: Application toward malaria elimination goals. Sci. Transl. Med..

[12] Kirillova A., Ionov L. (2019). Shape-changing polymers for biomedical applications. J. Mater. Chem. B.

[13] Kirtane A.R., Abouzid O., Minahan D., Bensel T., Hill A.L., Selinger C., Bershteyn A., Craig M., Mo S.S., Mazdiyasni H. (2018). Development of an oral once-weekly drug delivery system for HIV antiretroviral therapy. Nat. Commun..

[14] Zhao W., Liu L., Zhang F., Leng J., Liu Y. (2018). Shape memory polymers and their composites in biomedical applications. Mater. Sci. Eng. C.

[15] Behl M., Lendlein A. (2007). Shape-memory polymers. Mater. Today.

[16] Gall K., Yakacki C.M., Liu Y., Shandas R., Willett N., Anseth K.S. (2005). Thermomechanics of the shape memory effect in polymers for biomedical applications. J. Biomed. Mater. Res. Part A.

[17] Melocchi A., Inverardi N., Uboldi M., Baldi F., Maroni A., Pandini S., Briatico-Vangosa F., Zema L., Gazzaniga A. (2019). Retentive device for intravesical drug delivery based on water-induced shape memory response of poly(vinyl alcohol): Design concept and 4D printing feasibility. Int. J. Pharm..

[18] Melocchi A., Uboldi M., Inverardi N., Briatico-Vangosa F., Baldi F., Pandini S., Scalet G., Auricchio F., Cerea M., Foppoli A. (2019). Expandable drug delivery system for gastric retention based on shape memory polymers: Development via 4D printing and extrusion. Int. J. Pharm..

[19] Bandari S., Nyavanandi D., Dumpa N., Repka M.A. (2021). Coupling hot melt extrusion and fused deposition modeling: Critical properties for successful performance. Adv. Drug Deliv. Rev..

[20] Casati F., Melocchi A., Moutaharrik S., Uboldi M., Foppoli A., Maroni A., Zema L., Neut C., Siepmann F., Siepmann J. (2020). Injection molded capsules for colon delivery combining time-controlled and enzyme-triggered approaches. Int. J. Mol. Sci..

[21] Kallakunta V.R., Sarabu S., Bandari S., Tiwari R., Patil H., Repka M.A. (2019). An update on the contribution of hot-melt extrusion technology to novel drug delivery in the twenty-first century: Part I. Expert Opin. Drug Deliv..

[22] Melocchi A., Uboldi M., Parietti F., Cerea M., Foppoli A., Palugan L., Gazzaniga A., Maroni A., Zema L. (2020). Lego-Inspired Capsular Devices for the Development of Personalized Dietary Supplements: Proof of Concept with Multimodal Release of Caffeine. J. Pharm. Sci..

[23] Melocchi A., Uboldi M., Cerea M., Foppoli A., Maroni A., Moutaharrik S., Palugan L., Zema L., Gazzaniga A. (2020). A Graphical Review on the Escalation of Fused Deposition Modeling (FDM) 3D Printing in the Pharmaceutical Field. J. Pharm. Sci..

[24] Melocchi A., Uboldi M., Briatico-Vangosa F., Moutaharrik S., Cerea M., Foppoli A., Maroni A., Palugan L., Zema L., Gazzaniga A. (2021). The Chronotopic™ System for Pulsatile and Colonic Delivery of Active Molecules in the Era of Precision Medicine: Feasibility by 3D Printing via Fused Deposition Modeling (FDM). Pharmaceutics.

[25] Parulski C., Jennotte O., Lechanteur A., Evrard B. (2021). Challenges of fused deposition modeling 3D printing in pharmaceutical applications: Where are we now?. Adv. Drug Deliv. Rev..

[26] Sarabu S., Bandari S., Kallakunta V.R., Tiwari R., Patil H., A Repka M. (2019). An update on the contribution of hot-melt extrusion technology to novel drug delivery in the twenty-first century: Part II. Expert Opin. Drug Deliv..

[27] Simões M.F., Pinto R.M.A., Simões S. (2021). Hot-Melt Extrusion: A Roadmap for Product Development. AAPS PharmSciTech.

[28] Agarwal T., Hann S.Y., Chiesa I., Cui H., Celikkin N., Micalizzi S., Barbetta A., Costantini M., Esworthy T., Zhang L.G. (2021). 4D printing in biomedical applications: Emerging trends and technologies. J. Mater. Chem. B.

[29] Fu P., Li H., Gong J., Fan Z., Smith A.T., Shen K., Khalfalla T.O., Huang H., Qian X., McCutcheon J.R. (2022). 4D printing of polymers: Techniques, materials, and prospects. Prog. Polym. Sci..

[30] Han D., Morde R.S., Mariani S., La Mattina A.A., Vignali E., Yang C., Barillaro G., Lee H. (2020). 4D Printing of a bioinspired microneedle array with backward-facing barbs for enhanced tissue adhesion. Adv. Funct. Mater..

[31] Uboldi M., Melocchi A., Moutaharrik S., Palugan L., Cerea M., Foppoli A., Maroni A., Gazzaniga A., Zema L. (2022). Administration strategies and smart devices for drug release in specific sites of the upper GI tract. J. Control. Release.

[32] Wang Y., Miao Y., Zhang J., Wu J.P., Kirk T.B., Xu J., Ma D., Xue W. (2018). Three-dimensional printing of shape memory hydrogels with internal structure for drug delivery. Mater. Sci. Eng. C.

[33] Inverardi N., Scalet G., Melocchi A., Uboldi M., Maroni A., Zema L., Gazzaniga A., Auricchio F., Briatico-Vangosa F., Baldi F. (2021). Experimental and computational analysis of a pharmaceutical-grade shape memory polymer applied to the development of gastroretentive drug delivery systems. J. Mech. Behav. Biomed. Mater..

[34] Uboldi M., Melocchi A., Moutaharrik S., Cerea M., Gazzaniga A., Zema L. (2021). Dataset on a Small-Scale Film-Coating Process Developed for Self-Expanding 4D Printed Drug Delivery Devices. Coatings.

[35] Kirtane A.R., Hua T., Hayward A., Bajpayee A., Wahane A., Lopes A., Bensel T., Ma L., Stanczyk F.Z., Brooks S. (2019). A once-a-month oral contraceptive. Sci. Transl. Med..

[36] Verma M., Vishwanath K., Eweje F., Roxhed N., Grant T., Castaneda M., Steiger C., Mazdiyasni H., Bensel T., Minahan D. (2019). A gastric resident drug delivery system for prolonged gram-level dosing of tuberculosis treatment. Sci. Transl. Med..

[37] Verma M., Chu J.N., Salama J.A., Faiz M.T., Gwynne F.E.D., Lopes A., Hess K., Soares V., Steiger C., McManus R. (2020). Development of a long-acting direct-acting antiviral system for hepatitis c virus treatment in a swine model. Gastroenterology.

[38] Allopurinol. https://www.drugbank.ca/drugs/DB00437.

[39] Kulkarni R.V., Mutalik S., Hiremath D. (2002). Effect of plasticizers on the permeability and mechanical properties of Eudragit^®^ films for transdermal application. Indian. J. Pharm. Sci..

[40] Thakral S., Thakral N.K., Majumdar D.K. (2013). Eudragit^®^: A technology evaluation. Expert Opin. Drug Deliv..

[41] Bajdik J., Pintye-Hódi K., Regdon G., Fazekas P., Szabó-Révész P., ErÞs I. (2003). The effect of storage on the behaviour of Eudragit^®^ NE free film. J. Therm. Anal. Calorim..

[42] Baert L., Remon J. (1993). Water vapour permeation of aqueous based ethylacrylate methylmethacrylate copolymer films. Int. J. Pharm..

[43] Semdé R., Karim A., Devleeschouwer M.J., Moës A. (2000). Jtudies of pectin HM/Eudragit^®^ RL/Eudragit^®^ NE film-coating formulations intended for colonic drug delivery. Int. J. Pharm..

[44] Caramella C., Ferrari F., Bonferoni M.C., Gazzaniga A., E Sangalli M., Conte U., Valserra M.D.B.D., Feletti F. (1989). Swelling-restricted minimatrices for controlled release of drugs. Preliminary in-vivo studies. Boll. Chim. Farm..

[45] Colombo P., Conte U., Gazzaniga A., Maggi L., Sangalli M., Peppas N., Lamanna A. (1990). Drug release modulation by physical restrictions of matrix swelling. Int. J. Pharm..

[46] Gazzaniga A., Sangalli M., Conte U., Caramella C., Colombo P., La Manna A. (1993). On the release mechanism from coated swellable minimatrices. Int. J. Pharm..

[47] Hung S.-F., Hsieh C.-M., Chen Y.-C., Wang Y.-C., Ho H.-O., Sheu M.-T. (2014). Characterizations of Plasticized Polymeric Film Coatings for Preparing Multiple-Unit Floating Drug Delivery Systems (muFDDSs) with Controlled-Release Characteristics. PLoS ONE.

[48] Sadeghi F., Shahabi M., Afrasiabi H. (2011). Comparison of physicomechanical properties of films prepared from organic solutions and aqueous dispersion of Eudragit RL. DARU J. Pharm. Sci..

